# An Enhanced Secure Identity-Based Certificateless Public Key Authentication Scheme for Vehicular Sensor Networks

**DOI:** 10.3390/s18010194

**Published:** 2018-01-11

**Authors:** Congcong Li, Xi Zhang, Haiping Wang, Dongfeng Li

**Affiliations:** 1 School of Traffic and Transportation, Beijing Jiaotong University, Haidian District, Beijing 100000, China; xizhang@bjtu.edu.cn; 2 Research and Development Department, Beijing Zhonghaiwenda Information Technology Company, Haidian District, Beijing 100000, China; whp@mail.ustc.edu.cn; 3 Electronic Transaction Cryptographic Application Group, State Cryptography Administration Office of Security Commercial Code Administration, Fengtai District, Beijing 100000, China; lidongfeng66@163.com

**Keywords:** authentication, identity-based, certificateless, vehicular sensor network (VSN)

## Abstract

Vehicular sensor networks have been widely applied in intelligent traffic systems in recent years. Because of the specificity of vehicular sensor networks, they require an enhanced, secure and efficient authentication scheme. Existing authentication protocols are vulnerable to some problems, such as a high computational overhead with certificate distribution and revocation, strong reliance on tamper-proof devices, limited scalability when building many secure channels, and an inability to detect hardware tampering attacks. In this paper, an improved authentication scheme using certificateless public key cryptography is proposed to address these problems. A security analysis of our scheme shows that our protocol provides an enhanced secure anonymous authentication, which is resilient against major security threats. Furthermore, the proposed scheme reduces the incidence of node compromise and replication attacks. The scheme also provides a malicious-node detection and warning mechanism, which can quickly identify compromised static nodes and immediately alert the administrative department. With performance evaluations, the scheme can obtain better trade-offs between security and efficiency than the well-known available schemes.

## 1. Introduction

According to a report by the World Health Organization (WHO), the total number of worldwide road traffic deaths caused by various traffic accidents is 1.25 million per year [1]. To manage increasingly heavy traffic scenarios and enhance driving safety, wireless sensor networks and smart devices have recently been implemented on a large scale in the transportation systems of many countries. As part of an intelligent transportation system (ITS), vehicle sensor networks (VSNs) provide a better resolution to traffic problems via the collection, processing and dissemination of traffic information within the scope of interconnected sensor nodes, which are mounted on vehicles and roadsides. The static wireless access nodes alongside the roads, which are called Road Side Units (RSUs), are used to provide communication to vehicles and infrastructure in their coverage area. VSNs involve different network modules, such as Wireless Access in Vehicular Environment (WAVE) [2]/Dedicated Short-Range Communication (DSRC), Wireless Fidelity (Wi-Fi) and the 4th Generation Communication System (4G)/Long Term Evolution (LTE) that work together. Among them, Vehicle-to-Vehicle (V2V) and Vehicle-to-Infrastructure (V2I) communications are two main forms of VSNs that use the DSRC protocol [3] and WAVE to perform their operations in collaboration. VSNs are rapidly changing and self-organizing with multiple-hops topologies over wireless links. Various wireless communication devices on vehicles broadcast traffic information to RSUs or other vehicles every 100–300 milliseconds according to the DSRC. Thus, it must take a short amount of time to deal with a message without delay for VSN entities.

The information among VSN entities include traffic conditions (e.g., road defects, congestion situations and temperature conditions, etc.) and vehicle conditions (e.g., location, speed, traffic status, etc.) [2]. These messages are indispensable for vehicles and infrastructure, such as traffic control centers, which use these messages to make critical decisions in an emergency situation. If an adversary modifies messages or inserts malicious messages to the network, it will result in traffic chaos or even accidents. Furthermore, DSRC/WAVE is inferior to other network modules in terms of security support [4]. DSRC is a wireless protocol that makes data to be easily monitored, altered and forged, including sensitive data concerning drivers’ privacy [5]. Therefore, to protect users’ privacy and information integrity in VSNs is important. In addition, RSUs are always deployed in an unattended environment. Hardware tampering occurs when the sensors and other on-board hardware RSUs are manipulated by adversaries [6]. Adversaries may capture and take control the RSUs via a physical attack and extract all cryptographic information from the compromised RSUs, and they may relocate a tampered RSU to launch a malicious attack [7] or make many clones from the tampered RSU. Therefore, resisting RSU compromise and replication attacks is a key consideration in the designed authentication. However, many existing secure schemes fail to withstand RSU compromise attacks.

This paper presents an enhanced identity-based (ID-based) certificateless authentication scheme to solve the aforementioned problems. The main contributions provided are as follows:

1. The proposed scheme is based on the certificateless public key cryptograph (CLPKC) [8], which can solve the certificate management problem in the public key infrastructure (PKI) [9] and the key escrow’ problem in identity-based encryption (IBE) [10,11]. The scheme use the elliptic curve multiplication instead of the bilinear pairing because that the relative computational costs of a pairing operation are approximately 20 times higher than that of an elliptic curve scalar multiplication [12]. In addition, this scheme supports batch authentication by simultaneously verifying several messages. Moreover, the proposed scheme is provably secure against the adaptive chosen message attack in the random oracle model as long as the computational elliptic curve discrete logarithm problem (ECDLP) is intractable.
2. In the scheme, an anonymous communication and conditional privacy-preserving authentication are supported to protect users’ privacy. Every user is issued a smart card with distinct pseudo identities, which are generated by trusted authorities (TAs) according to user’s actual identity and secret information. The user’s actual identity can be uniquely revealed by the TA when necessary.
3. The proposed scheme uses a position-based authentication scheme to reduce the possibility of RSU capture attacks. The proposed scheme also provides a compromised-RSU detection and alarm mechanism to identify misbehaving RSUs and immediately alert the traffic administrative department.

## 2. Related Work 

In this section, we provide a brief summary of the related literature focused on authentication schemes in VSNs. Many authentication schemes have been proposed in recent years, and most of them are certificate-based or ID-based authentication schemes. Paruchuri et al. [13] proposed a certificate-based scheme, which provides anonymous authentication and location privacy using a smart card that stores the session keys of RSUs. However, this scheme fails to support V-to-V authentication. The RSUs and vehicles require additional computations to verify the certificates issued by the TA. In addition, each on-board unit (OBU) stores many session keys from different RSUs. And during the authentication process, the encrypted message is transmitted to identify the owner of the session key to be decrypted, which is inefficient for VSN authentication. Finally, if one RSU is compromised, then the stored session keys in the RSU, including the session keys of neighboring RSUs, are leaked.

Almeida et al. [14] proposed a PKI-related key distribution protocol for VSNs that alleviates the burden of traditional PKI authentication schemes. However, many different keys are stored in each vehicle, and when a node is compromised, it will trigger a key revocation in a distributed fashion, which may cause an undesirable communication overhead. In addition, the PKI-based authentication mechanisms require additional computational overhead to verify the certificates of others.

To improve the scalability of certificate-based authentication schemes for VSNs, Calandriello [15] proposed a pseudonym-based authentication scheme to achieve efficiency and robustness. This scheme authorizes each OBU to generate its own pseudonyms without affecting the system security. However, each mobile node (vehicle) preloads many pseudonyms and related certificates in the story, which uses a considerable amount of memory. During a time period of τ, the scheme can also suffer from a tracking attack if the signature ${Cert}_{CA}^{H}\left(K_{v}^{i}\right)$ is unchanged. Moreover, the scheme does not address certificate revocation.

Zhang et al. [16] proposed an RSU-aided message authentication scheme in which a vehicle obtains a symmetric key from a RSU and communicates with other vehicles using a keyed hash message authentication code (HMAC). However, the scheme is fully relies on RSUs. If one RSU is controlled or compromised, the scheme will collapse.

Because of the certificate management problem, an ID-based scheme is a more precise replacement for the PKI-based scheme for vehicular-network applications [17]. Authentication schemes that use IBE, which was proposed by Shamir [10] in 1984 have been implemented in VSNs. Chim [18] proposed an ID-based authentication scheme with batch verification based on the above bilinear pairings for secure V-to-I communications. This scheme has lower communication costs than previously proposed ID-based schemes. However, Horng et al. [19] found that Chim’s scheme was vulnerable to impersonation attacks, in which a malicious vehicle can impersonate a valid vehicle and send fake messages to the RSUs or other vehicles. Horng et al. provided a secure scheme that overcame the weaknesses of the scheme in [18]. However, because the computational costs of one pairing operations are at least three times higher than that of a one point multiplication operation [20], these two schemes require heavy computational costs in the signature verification phase and are not suitable in rapidly changing networks. Furthermore, these mechanisms are only considered suitable for private networks [21] because of the key escrow problem based on IBE.

In 2003, Al-Riyami and Paterson [8] developed the concept of CLPKC. In this scheme, the full private key consists of two parts: the partial private key generated by the Private Key Generator (PKG) and the secret key selected by the user. Therefore, this scheme can solve the certificate management problem in PKI and the key escrow problem in IBE. Shim [22] proposed a secure conditional privacy-preserving authentication scheme (CPPA) using a pseudo-identity-based signature (IBS) scheme without using the MaptoPoint hash function [23]. This scheme achieves anonymous authentication, message integrity, traceability, and unlinkability, and it also maintains a balance between privacy and traceability. However, Liu [24] noted that Shim’s scheme could not be normal existential unforgeable against adaptive chosen-identity and chosen-message attacks. Pankaj [25] proposed an efficient certificateless signature scheme in HWSN. However, the scheme is lack of traceability and preserving identity privacy. Also, it suffered from a high overhead using bilinear pairing operation.

To reduce the authentication time and improve the computational efficiency for VSNs, He et al. [26] propose an ID-based CPPA scheme for VSNs based on the Elliptic Curve Cryptography (ECC), which satisfies security and privacy requirements. The scheme is more efficient than previously proposed schemes for VSNs. However, this scheme heavily relies on a tamper-proof hardware device in which an important master secret key is preloaded for each vehicle. If the master secret key is extracted by adversaries though side-channel attacks, such as power analyses and laser scanning [22], all malicious messages generated by the adversaries can be successfully verified and the entire system will be compromised. Lo et al. [27] proposed a faster ID-based scheme for VSNs based on ECC without using the special MaptoPoint hash function, which is efficient and consumes more computing time. This scheme also supports the batch signature and conditional privacy-preserving authentication; however, it is significantly dependent on secure communication channels. In the particle scenario, the vehicle-specific information is easily collected from overhearing the wireless network [7]. From the implementation perspective, the scheme has high costs and lacks of scalability. In addition, the schemes [26,27] suffered from privileged insider attacks in the PKG. If an adversary obtains the private key of one user issued by the PKG, he can easily forge a valid signature.

## 3. Background

In this section, we briefly introduce the network model and adversary model of our scheme.

### 3.1. Network Model

The proposed scheme applies a two-layer network model. The upper layer consists of the PKG, TA and a traffic information service center. The bottom layer includes vehicles equipped with wireless communication device and RSUs, which can communicate with one another using the DSRC/WAVE protocol.

Here, we should consider two application scenarios according to different locations of RSUs. First, RSUs are built on main roadways, which are the focus of most other schemes. The infrastructure and RSUs communicate through secure channels, such as the transport layer security protocol via wired connections [19]. Second, RSUs are deployed in unattended environments, such as highway roads. Thus, the cost of constructing optic and electric composite cables to provide power and communication between the RSUs and the infrastructure is high. In the second scenario, we deploy RSUs with batteries and short wireless communication ranges. Users can contact RSUs via single-hop or multi-hop communication, which is more robust and suitable for the second scenarios.

**Figure 1:**
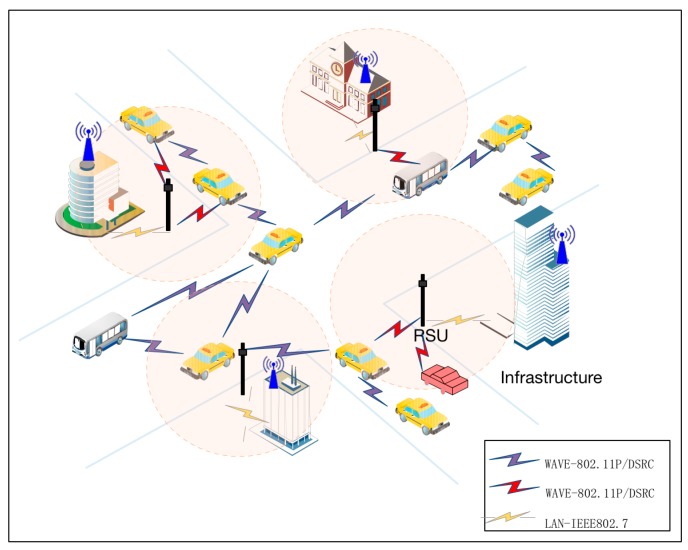
Network architecture on the main roadways.

**Figure 2:**
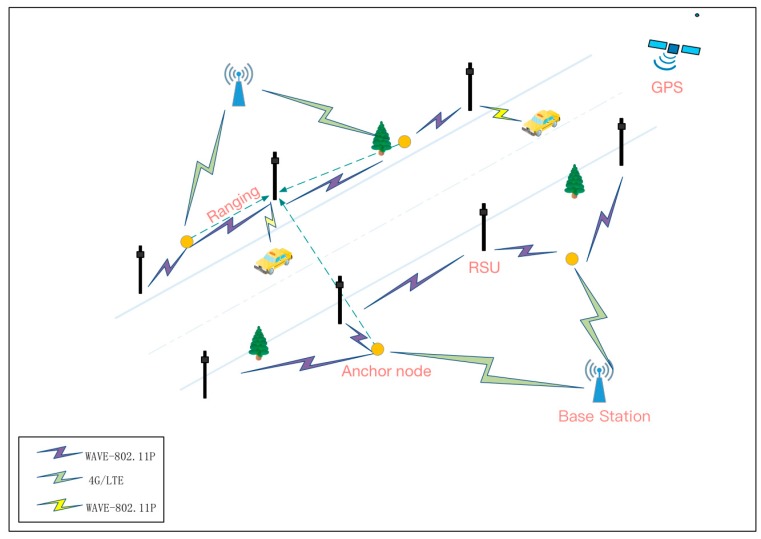
Network architecture in a desolate environment.

The two scenarios are shown in the Figure 1 and Figure 2.

**TA:** The TA registers the drivers and generates pseudo identities for valid users. The TA is the only party that can trace the vehicle and reveal the identities from the signers. The TA cannot be compromised and is fully trusted by all parties in the system.

**PKG:** The PKG is a trusted third party that generates partial private keys for the signers.

**RSUs:** RSUs are distributed along road sides equipped with an on-board sensory, processing, and wireless access point, and they are mainly used to verify the messages and transfer data among the vehicles and infrastructure in its coverage area, such as the traffic information service center, TA and PKG.

**Vehicle:** All vehicles are equipped with card reader, on-board sensory, processing, and wireless communication modules. All users who want to access the services from the VSNs will be issued a smart card with system parameters, which can help the TA to track the behaviors back to the owner of the smart card instead of the car. Smart card technology conforms to international standards (ISO/IEC 7816 and ISO/IEC 14443) [28,29]. With an embedded microcontroller, each smart card can store large amounts of data, and they have the computing ability to perform on-card functions (e.g., signature and authentication). The smart card can interact with card reader, which is mounted on the car. The communication protocol with neighboring vehicles and RSUs is 5.9-GHz DSRC [3] IEEE 802.11p.

**Anchor nodes:** In Figure 2, to prevent adversaries from inserting malicious nodes into the networks, the key point of our approach is to deploy certain anchor nodes with higher processing capabilities and a global position system (GPS) receiver. These nodes can help the system to reduce the possibility of static nodes (RSUs and anchor nodes) compromise attacks and immediately detect nearby controlled nodes using our method. We elaborate on the function of anchor nodes in Section 4.3.

### 3.2. Adversary Model

In reality, all communication channels among VSN entities are not explicitly secure. In Lo’s scheme, every transmit channel is assumed to be secure without considering this fact. In this paper, we assume that the communication channels are public and adversaries can conduct attacks, such as eavesdropping, insider attacks, stolen smart-card attacks and impersonation attacks, in which adversaries attempt to impersonate a legitimate user or a node. In addition, the adversary can conduct a physical attack on static nodes (RSUs and anchor nodes) and retrieve secret information and stored data from them particularly in an unwatched location. In further attacks, the adversary attempts to replicate the controlled nodes, deploy them in other places and manipulate the network with the clones or captured nodes.

## 4. Proposed Scheme

In this section, we proposed an enhanced ID-based certificateless authentication scheme based on the modification of the original CLPKC mechanism [8]. The scheme supports the V2I and V2V communication, and it consists of five phases: System Initialization, Register, Login, Signing and Verification. The symbols of our scheme are described in Table 1.

### 4.1. System Initialization

The PKG generates system parameters via running following steps. First, the PKG chooses a k-bit *prime* number n and generates the tuple $\left\{F_{n},\;E\left(F_{n}\right),\;G_{q},\;P\right\}$. Then the PKG picks a random number $s\in Z_{q}^{*}$ as its private key and computes $P_{PKG}=s\cdot P$. Furthermore, the PKG determines four one way hash functions: $h_{0}:{\left\{0,\;1\right\}}^{*}\to Z_{q}^{*}$, $h_{1}:{\left\{0,\;1\right\}}^{*}\times G_{q}\times {\left\{0,\;1\right\}}^{*}\to Z_{q}^{*}$, $h_{2}:G_{q}^{2}\times {\left\{0,\;1\right\}}^{*}\times {\left\{0,\;1\right\}}^{*}\to Z_{q}^{*}$, $h_{3}:{\left\{0,\;1\right\}}^{*}\times G_{q}^{2}\times {\left\{0,\;1\right\}}^{*}\times G_{q}^{\;}\times {\left\{0,\;1\right\}}^{*}\to Z_{q}^{*}$. The TA also selects a random $r\in Z_{q}^{*}$ as its private key and computes $P_{TA}=r\cdot P$. At last, the PKG publish system parameters $Z=\left\{F_{n},\;E\left(F_{n}\right),\;G_{q},\;P,\;P_{PKG},\;P_{TA},\;h_{0},\;h_{1},\;h_{2},\;h_{3}\right\}$. The PKG and TA keep s and r secret, respectively.

### 4.2. Vehicle to RSU (the RSU Verifies the Vehicle)

#### 4.2.1. Register

Every user who wants to access the services from VSNs is issued a smart card with system parameters offline from the TA at first. Note that the user must disclose his valid credentials such as ID card or driving license to the TA to get the smart card. The user’s credential number (the real identity ID of the user) is input to the smart card by the TA and will be recorded in the list of TA. In the beginning of the smart card activation, the user inserts his smart card into a card reader mounted on a car, and input his real identity ${ID}^{\prime }$ and password PW. Note that the real identity is registered in the TA offline and can uniquely identify the user.

Upon receiving the ${ID}^{\prime }$ and PW, in which $ID\in Z_{q}^{*}$ and $PW\in Z_{q}^{*}$, the smart card compares ${ID}^{\prime }$ with the stored one. If true, the smart card calculates $h_{0}\left(PW⊕b\right)$ and $h_{0}\left(ID\right)$, in which the $b\in Z_{q}^{*}$ is an arbitrary number and the length of b is enough large. Then the smart card selects a random number $d_{1}\in Z_{q}^{*}$ as the user’s secret value and generates the public key $P_{1}=d_{1}\cdot P$. Subsequently, the smart card sets $s_{1}{=h}_{0}\left(PW⊕b\right)⊕ID$ and $s_{2}=s_{1}+d_{1}$. The smart card encrypts $\left\{ID,h_{0}\left(PW⊕b\right),P_{1}\right\}$ using the TA’s public key and sends it to the TA.

Upon receiving the register request, the TA decrypts it using the TA’s private key r and checks whether the ID is legal, and if so, the TA will make m pseudo identities for the user. The TA computes:

(1) $${PID}_{1,i}=r\times h_{1}\left(Enc_{P_{TA}}(ID)⊕h_{0}\left(PW⊕b\right){||P}_{1}||T\right)+n_{i}\;\mathrm{mod}q\;,N_{i}=n_{i}\cdot P,\left(i=1\ldots m\right),$$

where $n_{i}\in Z_{q}^{*}$ is a random number, $T\in Z_{q}^{*}$ is the expiration date of the $P{ID}_{1}$ and m is the number of PIDs. For convenience, we set $\left\{Enc_{P_{TA}}\left(ID\right)⊕h_{0}\left(PW⊕b\right)\right\}=H_{1}$. The TA encrypts these PIDs $\left\{PID_{1},\;H_{1},\;N,\;T\right\}$ using $P_{1}$ and sends it to the smart card. Note that the TA stores the $Enc_{P_{TA}}\left(ID\right)$ instead of the ID to prevent stolen ID list attacks. The TA stores the $\left\{PID,\;Enc_{P_{TA}}\left(ID\right),\;{\;h}_{0}\left(PW⊕b\right),\;H_{1},\;N\right\}$ in its memory.

When receives $Enc_{P_{1}}\left\{{PID}_{1},\;H_{1},\;N,\;T\right\}$, the smart card decrypts and checks them via running ${PID}_{1,i}\cdot P=P_{TA}\cdot h_{1}\left(H_{1}||P_{1}||T\right)+N_{i},\;\left(i=1\ldots m\right)$. If the equations hold, which mean that adversaries do not tamper the pseudo identities, and the smart card calculates ${PID}_{i}={PID}_{1,i}+d_{1},\;\left(i=1\ldots m\right)$. Otherwise, reject the ${PID}_{1}$. Here, every PID. is generated as a combination of secret value of the TA and the user-chosen secret. Thus, adversaries cannot forge the valid PID without the user-chosen secret $d_{1}$. Subsequently, the smart card sends the tuples $\left\{{PID,\;H}_{1},\;P_{1},\;N,\;T\right\}$ to the PKG through a public channel.

Upon receiving the partial-secret-key request $\left\{{PID,\;H}_{1},\;P_{1},\;N,\;T\right\}$, the PKG validates the PIDs by checking whether the following equations:

(2) $${PID}_{i}\cdot P=P_{TA}\cdot h_{1}\left(H_{1}{||P}_{1}||T\right)+N_{i}+P_{1},\left(i=1\ldots m\right)$$

hold within the validity of T. If yes, then the PKG generates partial secret keys for users as below:

$$P_{2,i}=k_{i}\cdot P$$

(3) $$d_{2,i}=k_{i}+h_{2}\left(P_{1},P_{2,i},{PID}_{i},T\right)\times s\;\mathrm{mod}\;q,\;\left(i=1\ldots m\right)$$

where $k_{i}\in Z_{q}^{*}$ is a random number. The PKG sends $\left\{{PID,\;P}_{2},\;d_{2}\right\}$ back to the smart card. Else, reject the partial-secret-key request.

Upon receiving the partial secret keys, the smart card checks the authenticity of $\left\{PID,\;P_{2},\;d_{2}\right\}$ via running:

(4) $$d_{2,i}\cdot P=P_{2,i}+h_{2}\left(P_{1},P_{2,i},{PID}_{i},T\right)\cdot P_{PKG},\;\left(i=1\ldots m\right).$$

**Figure 3:**
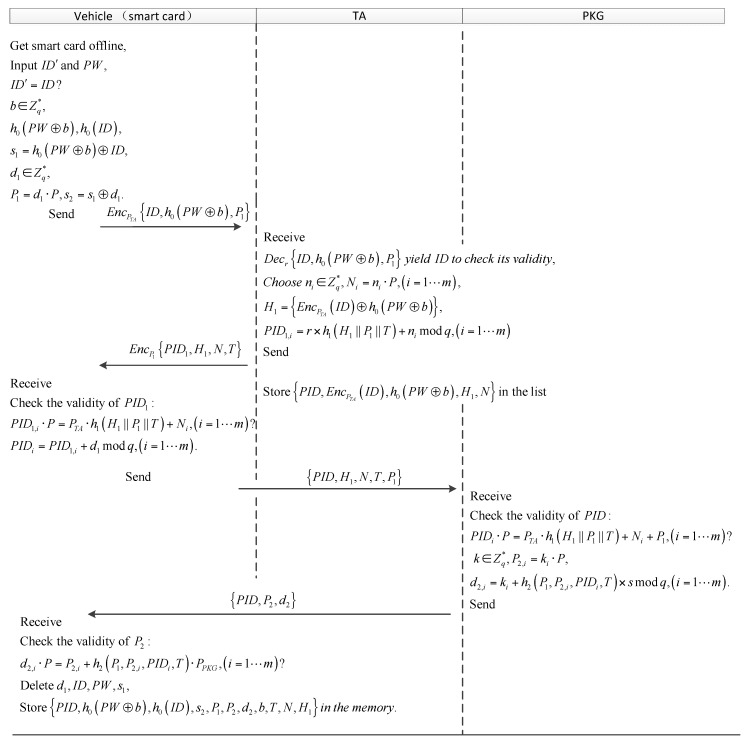
The vehicle to RSU (vehicle) registration process.

If the equations hold, which imply that the $\left\{P_{2},d_{2}\right\}$ are generated by the PKG. Otherwise, reject them. Then the smart card stores $\left\{PID,\;h_{0}\left(PW⊕b\right),\;h_{0}\left(ID\right),\;s_{2},P_{1},\;P_{2},\;d_{2},\;b,\;T,\;N,\;H_{1}\right\}$ in the memory and deletes $d_{1}$, ID, PW, $s_{1}$ to prevent smart card compromise attacks. The steps of the phase are depicted in Figure 3.

#### 4.2.2. Login and Message Signing

The user inserts his smart card into a card reader, and inputs ${ID}^{\prime }$ and ${PW}^{\prime }$. Then the smart card compares $h_{0}\left({PW}^{\prime }⊕b\right)$ and $h_{0}\left({ID}^{\prime }\right)$ with the stored ones in it. If true, the smart card computes $s_{1}^{\prime }=h_{0}\left({PW}^{\prime }⊕b\right)⊕{ID}^{\prime }$ and $d_{1}^{\prime }=s_{1}^{\prime }⊕s_{2}$, and checks the validity period of PIDs, then performs the following operations. Otherwise, reject the request. The smart card deletes the ${ID}^{\prime }$, $s_{1}^{\prime }$ and ${PW}^{\prime }$.

1. Generate a traffic-related message M, then pick a random number $l\in Z_{q}^{*}$ and calculate L=l·P to give a freshness.
2. Choose a ${PID}_{i}$ and its corresponding $d_{2,i}$, and calculate:
  (5) $$v=l+d_{2,i}+d_{1}^{\prime }\times h_{3}\left({PID}_{i},\;P_{1},\;P_{2,i},\;M,\;L,\;time\right)\;\mathrm{mod}q,$$
  where time is the current timestamp of the users’ system.
3. Send $\left\{{PID}_{i},\;P_{1},\;P_{2,i},\;M,\;L,\;T,\;v,\;time\right\}$ to another VSN entities.

#### 4.2.3. Verification

This phase is invoked when the verifier (a vehicle or RSU) receives the information $\left\{{PID}_{i},\;P_{1},\;P_{2,i},\;M,\;L,\;T,\;v,\;time\right\}$ at the time ${time}^{*}$, it uses the system parameters $Z=\left\{F_{n},\;E\left(F_{n}\right),\;G_{q},\;P,\;P_{PKG},\;P_{TA},\;h_{0},\;h_{1},\;h_{2},\;h_{3}\right\}$ to perform the following steps:

1. Validate the freshness of ${time}^{*}$. If ${time}^{*}-time\leq \cdot T$, then the verifier proceeds to the next step, else rejects the request, where ·T indicates the valid time interval.
2. Then the verifier checks the expire time T of ${PID}_{i}$.
3. The verifier checks the equation:
  (6) $$v\cdot P=L+P_{2,i}+h_{2}\left(P_{1},\;P_{2,i},\;{PID}_{i},\;T\right)\cdot P_{PKG}+P_{1}\cdot h_{3}\left({PID}_{i},\;P_{1},\;P_{2,i},\;M,\;L,\;time\right)$$

If it holds, the verifier accepts the M, else outputs “invalid”.

**Figure 4:**
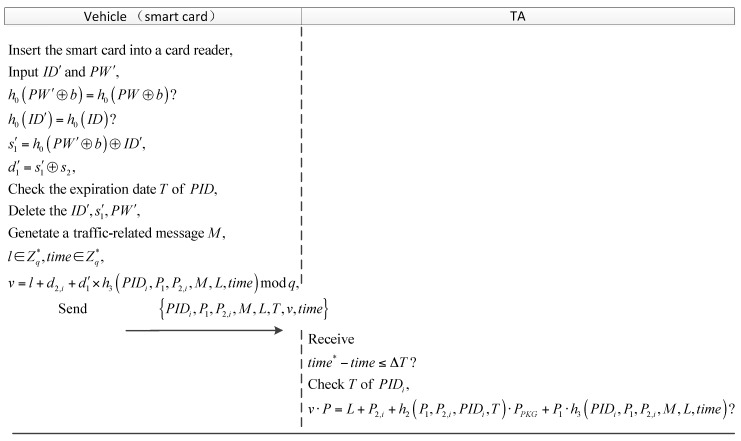
The vehicle to RSU (vehicle) authentication process.

After the user log out, the smart card delete the $d_{1}$ from its memory to prevent stolen smart card attacks. The steps of the phase are depicted in Figure 4.

#### 4.2.4. Batch Verification

To enhance the effectiveness of the message verification, we require that vehicles or RSUs can aggregate n signatures into a single one and handle it at the same time. In the batch verification scheme, if one of the signatures is invalid, all signatures will be dropped or rejected. The proposed scheme supports batch verification. When the verifier receives numbers of requests, denoted as $\left\{{PID}_{i,x},\;P_{1,x},\;P_{2i,x},\;M_{x},\;L_{x},\;T_{x},\;v_{x},\;{time}_{x}\right\},\;\left(x=1\cdots n\right)$, it adds several random numbers to quickly detect which message is invalid in the batch. The concept is regarded as an efficient method in the batch verification [24].

The verifier checks the following equation:

(7) $$\begin{array}{c} \left(\overset{n}{\underset{x=1}{\sum }}y_{x}v_{x}\right)\cdot P=\overset{n}{\underset{x=1}{\sum }}y_{x}L_{x}+\overset{n}{\underset{x=1}{\sum }}y_{x}P_{2i,x}+\left(\overset{n}{\underset{x=1}{\sum }}y_{x}h_{3,x}\left({PID}_{i,x},\;P_{1,x},\;P_{2i,x},\;M_{x},\;L_{x},\;{time}_{x}\right)\cdot P_{1,\;x}\right)\\ +\left(\overset{n}{\underset{x=1}{\sum }}y_{x}h_{2,x}\left(P_{1,x},\;P_{2i,x},\;{PID}_{i,\;x},\;T_{x}\right)\right)\cdot P_{PKG}, \end{array}$$

where $y_{x}\left(x=1\cdots n\right)$ are small random numbers.

If the equation holds, than the verifier accepts these messages, else detects the invalid messages and rejects them.

### 4.3. RSU to Vehicle (the Vehicle Verifies the RSU) 

In this subsection, we use a position-based authentication method to reduce the possibility of node capture attacks.

As indicated in Section 3.1, there are two types of nodes. The anchor nodes and normal RSUs. The difference between them is that the anchor nodes obtain their position with the help of the built-in GPS receivers, whereas they are unknown for the RSUs. The anchor nodes have more computation and energy power than that of the RSUs. The anchor node has two main functions. First, it broadcasts its position in real time to help nearby RSUs calculate their coordinates. Second, it can immediately detect abnormal RSUs inside its range.

We implement an efficient approach based on the Received Signal Strength Indication (RSSI) combined with the centroid algorithm [30], which is high accurate to obtain the position. RSSI-based location schemes are the most prevalent ones due to their easier implementation and less complexity [31], especially for the energy-constrained nodes. Therefore, with this method, if a RSU is captured and moved to another location, it will fail to be verified because that the new position incorporated in the signature is changed. Furthermore, the anchor node can immediately detect abnormal RSUs via comparing the two locations, and the first one is obtained by the GPS and the other one is calculated by nearby RSUs. If the value does not change a lot within the measurement uncertainties, then the nearby RSUs are valid, else abnormal RSUs must be surrounding the anchor node, say get captured, replicated, or moved by adversaries, and the anchor nodes will immediately alert to the PKG.

#### 4.3.1. Initialization

Every RSU is preloaded a legitimate ${ID}_{R1}$ assigned by the PKG, which is stored in its tamper-proof device. Every anchor node is assigned a ${ID}_{c}$ and deployed in its pre-setup position by the PKG. After deployment, the RSU receives the position information from nearby anchor nodes at the first time. The details of the information are as follows:

(8) $$\begin{array}{c} L_{c1}=\left\{{ID}_{c1},\;P_{c1},\;\left(x_{c1},\;y_{c1}\right)\right\}\\ L_{c2}=\left\{{ID}_{c2},\;P_{c2},\;\left(x_{c2},\;y_{c2}\right)\right\}\\ L_{c3}=\left\{{ID}_{c3},\;P_{c3},\;\left(x_{c3},\;y_{c3}\right)\right\}\\ \vdots \\ L_{ci}=\left\{{ID}_{ci},\;P_{ci},\;\left(x_{ci},\;y_{ci}\right)\right\}, \end{array}$$

where $L_{ci}$ denotes the position information broadcasted by the anchor node, and $P_{ci}=d_{ci}\cdot P$ is its public key, in which $d_{ci}\in Z_{q}^{*}$ is a random number as its secret key, and $\left(x_{ci},\;y_{ci}\right)$ is the current coordinates measured by the GPS.

The RSU computes its current coordinates $\left(x_{R},\;y_{R}\right)$ according to the any of three coordinates of anchor nodes through centroid algorithm based on the RSSI [30] mentioned above and sets ${ID}_{R2}=h_{0}\left(\left(x_{R},y_{R}\right)\right)$. Subsequently, the RSU chooses a random number $d_{R1}\in Z_{q}^{*}$ as its secret key, and sets $\;P_{R1}=d_{R1}\cdot P$. Then the RSU set $S_{d_{R1}}={Sign}_{d_{R1}}\left\{{ID}_{R1}∥{ID}_{R2}∥L_{c1}∥L_{c2}∥L_{c3}∥\cdots ∥L_{cn}∥P_{R1}\right\}$ signing with the secret key $d_{R1}$ and encrypts the tuple $\left\{S_{d_{R1}}∥{ID}_{R1}∥{ID}_{R2}∥L_{c1}∥L_{c2}∥L_{c3}∥\cdots ∥L_{cn}∥P_{R1}\right\}$ using the public key of the PKG, and the RSU sends it to the PKG.

Upon receiving the tuple, the PKG decrypts it and verifies the signature. Then the PKG compares the $L_{ci}$ and ${ID}_{R1}$ with the stored list to make sure that they are legitimate ones without being modified at the initialization step.

The PKG generates the partial secret key for RSUs as follows:

$$P_{R2}=k_{R}\cdot P$$

(9) $$d_{R2}=k_{R}+h_{2}\left(P_{R1},\;P_{R2},\;{ID}_{R2},\;t\right)\times s\;\mathrm{mod}\;q,$$

where $k_{R}\in Z_{q}^{*}$ is a random number and t is the expiration date of $d_{R2}$, then the PKG sends $\left\{{ID}_{R2},\;P_{R2},\;d_{R2},\;t\right\}$ back to the RSU.

The PKG calculates ${ID}_{R}={ID}_{R1}⊕{ID}_{R2}$ and $h_{0}\left({ID}_{R1}\right)$ in the next step, and deletes ${ID}_{R1}$ and ${ID}_{R2}$ from the list to avoid the stolen ID list attacks.

Upon receiving the $\left\{{ID}_{R2},\;P_{R2},\;d_{R2},\;t\right\}$, the RSU verifies the validity of $d_{R2}$ via checking the equation $d_{R2}\cdot P=P_{R2}+h_{2}\left(P_{R1},\;P_{R2},\;{ID}_{R2},\;t\right)\cdot P_{PKG}$. If the equation holds, then it accepts the $d_{R2}$, else it applies the PKG for the partial secret key again. Then the RSU calculates the short-term pairwise encryption keys:

(10) $$\begin{array}{c} k_{1}=d_{R1}\cdot P_{c1}\\ k_{2}=d_{R1}\cdot P_{c2}\\ k_{3}=d_{R1}\cdot P_{c3}\\ \vdots \\ k_{n}=d_{R1}\cdot P_{cn} \end{array}$$

between the anchor nodes and RSUs.

#### 4.3.2. Message signing

The RSU picks a random number $l_{R}\in Z_{q}^{*}$ and sets $L_{R}=l_{R}\cdot P$, and it receives the location information from the anchor nodes and calculates the current coordinates $\left(x_{R}^{\prime },\;y_{R}^{\prime }\right)$ by the location algorithm. Let B be a position tolerance value, and the RSU should compare the new coordinates $\left(x_{R}^{\prime },\;y_{R}^{\prime }\right)$ with the previous one. If the distance $d=\sqrt{{\left(x_{R}^{\prime }-x_{R}\right)}^{2}+{\left(y_{R}^{\prime }-y_{R}\right)}^{2}}\leq B$, then the RSU sets ${ID}_{R2}^{\prime }={ID}_{R2}$, else renews the value ${ID}_{R2}={ID}_{R2}^{\prime }$.

Then the RSU calculates:

(11) $$v_{R}=l_{R}+d_{R2}+d_{R1}{\times h}_{3}\left({ID}_{R2}^{\prime },\;P_{R1},\;P_{R2},\;M,\;L_{R},\;time\right)\;\mathrm{mod}\;q,$$

in which time is the current timestamp of the RSU’s system and M is a traffic-related message.

Send $\left\{\left(x_{R}^{\prime },\;y_{R}^{\prime }\right),\;{ID}_{R2}^{\prime },\;P_{R1},\;P_{R2},\;M,\;L_{R},\;t,\;time,\;v_{R}\right\}$ to another VSN entities.

#### 4.3.3. Verification

When verifier such as a vehicle, anchor node or a RSU receives $\left\{(x_{R}^{\prime },\;y_{R}^{\prime }),\;{ID}_{R2}^{\prime },\;P_{R1},\;P_{R2},\;M,\;L_{R},\;t,\;time,\;v_{R}\right\}$ at time ${time}^{*}$, it firstly checks the fressness of ${time}^{*}$ and the expiration time t of the partial private key $d_{R2}$.

The verifier checks the equation:

(12) $$v_{R}\cdot P=L_{R}+P_{R2}+h_{2}\left(P_{R1},\;P_{R2},\;{ID}_{R2}^{\prime },\;t\right)\cdot P_{PKG}+P_{R1}\cdot h_{3}\left({ID}_{R2}^{\prime },\;P_{R1},P_{R2},\;M,\;L_{R},\;time\right)$$

If the equation holds, the verifier accepts the message M.

Upon receiving the signed message, the nearby anchor nodes perform the different steps inside their range, which firstly check the list and if there is no short-term pairwise encryption key $k_{i}$ with the RSU, the nodes calculate the $k_{i}$ via $k_{i}=d_{cj}\cdot P_{R1,i}$. Furthermore, the anchor nodes recount their coordinates according to ${ID}_{R2}^{\prime }$ and compare with previous ones. If the value significantly changes, then the RSU is abnormal, which is forged by the adversaries, and the anchor node generates an alert that is sent to the PKG. To prevent location information tampering attacks by adversaries, the anchor node encrypts its location using $k_{i}$ and broadcasts $L_{cj}=\left\{{ID}_{cj},\;P_{cj},\left(x_{cj},\;y_{cj}\right),\;h_{k_{i}}\left(\left(x_{cj},\;y_{cj}\right)\right)\right\}$ to RSUs next time.

**Figure 5:**
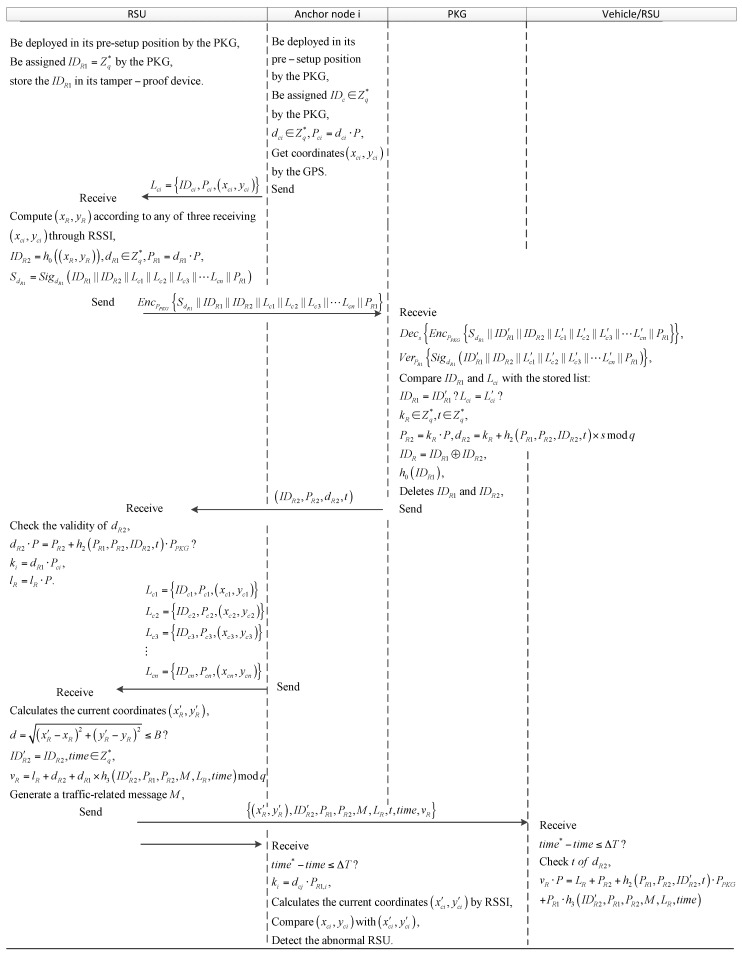
The RSU to vehicle (RSU) authentication process.

Here, $h_{k_{i}}\left(\left(x_{cj},\;y_{cj}\right)\right)$ is an encrypted digest called HMAC, which is viewed as a hash function and encrypted by the session key $k_{i}$ shared between the two entities. The steps of the phase are depicted in Figure 5.

The proposed scheme also supports the batch verification, and the process is as same as the one in Section 4.2.4.

### 4.4. Key Update

To prevent key compromise attacks for a long time, key update periodically is required. We divide this section into two parts, the user-key update and the RSU-key update:

(1) Updating a user’s $P_{W}^{i}$. This function is invoked whenever the user wants to update his password of the smart card. First, the user inserts his card into a card reader and inputs the original ${ID}_{i}^{\prime }$ and ${PW}_{i}^{\prime }$. Then, the smart card calculates $h_{0}\left({PW}_{i}^{\prime }⊕b\right)$ and $h_{0}\left({ID}_{i}^{\prime }\right)$, and it checks whether $h_{0}\left({PW}_{i}^{\prime }⊕b\right)=h_{0}\left({PW}_{i}⊕b\right)$ and $h_{0}\left({ID}_{i}^{\prime }\right)=h_{0}\left({ID}_{i}\right)$. If yes, the user will be allowed to input his new password ${PW}_{i}^{*}$ and proceed to the next step, else abort. Subsequently, the smart card recounts $h_{0}\left({PW}_{i}^{*}⊕b_{\prime }\right)$ and $h_{0}\left({ID}_{i}^{*}\right)$, in which $b_{\prime }$ is a new arbitrary number picked by the smart card, then it updates $s_{1}^{*}=h_{0}\left({PW}_{i}^{*}⊕b_{\prime }\right)⊕{ID}_{i}^{*}$ and $s_{2}^{*}=s_{1}^{*}⊕d_{1}^{*}$, in which $d_{1}^{*}$, as the user’s new secret value, is a random number reselected by the smart card. The subsequent steps are as same as the ones in Section 4.2.1.
(2) Updating a user’s pseudo identities and partial secret keys. User’s pseudo identities PIDs and partial secret keys share a same refresh cycle T. Every PID is appended an expiring time T by the TA for all users. Note that the period of T, which is relative to the key length and the complexity of circumstances, can be fixed by the administrator of the TA. When a user logs in the smart card, it firstly checks the T of PIDs, if the T is out of the valid date, the smart card terminates the following authentication process and informs the user to update the PIDs and related the partial secret keys. Note that any user cannot change the valid date T without the secret key of the PKG.
(3) Updating a RSU’s partial secret key. In general, the process is as same as the one of user’s. In addition, the updating phase is invoked when a valid RSU is authorized by the PKG to change its position. After deploying in a new location, the RSU will lunch a new handshake with the PKG to get a new partial secret key as same as the one in Section 4.3.1. Any node that attempts to change the position and tries to get a new key without the PKG’s authority is considered as a malicious node.

## 5. Security Proof 

In this section, we design four experiments to prove the security of the proposed scheme.

### 5.1. Experiment 1

We divide the kinds of adversaries into three according to their attack abilities in the scheme. The Type Ⅰ adversary A1 is not able to access the master key of the PKG or the secret keys of users. The Type Ⅱ adversary A2 represents a curious PKG who can access the master key of the PKG and obtain the partial secret keys of users but cannot forge secret keys of users. The type Ⅲ adversary A3 represents a malicious PKG who not only obtains the master key of the PKG but also has the right to generate secret keys of users at will, but the keys are different from that of users.

**Theorem** **1.** *We will demonstrate that our scheme is unforgeable against adaptive chosen message attacks of the adversary A1 under the random oracle due to the intractability of ECDLP*.

**Proof.**  There are two roles in the game, the challenger C and the adversary A. C can solve the ECDLP problem with a non-negligible probability by running A as a subroutine. For instance, when C receives a problem Q=s·P, $s\in Z_{q}^{*}$ is a random number, to calculates s is his target. C picks ${PID}^{*}$ as a challenged identity and sets system public key $P_{PKG}=x\cdot P$, then C sends the system params $\left(p,q,P,P_{PKG},h_{1},h_{2}\right)$ to the adversary A1. We show the process, in which C can break ECDLP by using the adversary A as follows. C maintains 4 lists $h_{1}^{list},\;h_{2}^{list},\;d_{1}^{list},\;d_{2}^{list}$, which are initially empty, and simulates oracles queried by A.

1. $h_{1}$ query. C maintains a list with the form of $\left({PID}_{i},\;P_{1i},\;P_{2i},\;T_{i},\;B_{i},\;coin\right)$. When A makes a query on $\left({PID}_{i},\;P_{1i},\;P_{2i}{,T}_{i}\right)$, if the list contains the tuple $\left({PID}_{i},\;P_{1i},\;P_{2i},\;T_{i},\;B_{i},\;coin\right)$ matched ${PID}_{i}$, C returns $B_{i}$ to A as a response. Otherwise, C chooses a random number $\mathrm{coin}\leftarrow_{R}\left\{0,1\right\}$ and sets Pr[coin=0]=δ, in which coin=0 means that this ${PID}_{i}$ is the challenged identity. Then C picks $B_{i}\leftarrow_{R}Z_{q}^{*}$ and sends $B_{i}=h_{1}\left({PID}_{i},\;P_{1i},\;P_{2i},\;T_{i}\right)$ to A as a response. C adds $\left({PID}_{i},\;P_{1i},\;P_{2i},\;T_{i},\;B_{i},\;coin\right)$ to $h_{1}^{list}$.
2. $h_{2}$ query. When A makes a query on $\left({PID}_{i},\;P_{1i},\;P_{2i},\;M_{i},\;L_{i},\;{time}_{i}\right)$, if the tuple $\left({PID}_{i},\;P_{1i},\;P_{2i},\;M_{i},\;L_{i},\;{time}_{i},\;D_{i}\right)$ exists in the list, then C sends it to A as a response. Otherwise, C picks a random $D_{i}\in Z_{q}^{*}$ and sets $D_{i}=h_{2}\left({PID}_{i},\;P_{1i},\;P_{2i},\;M_{i},\;L_{i},\;{time}_{i}\right)$, and C sends it to A as a response. C adds $\left({PID}_{i},\;P_{1i},\;P_{2i},\;M_{i},\;L_{i},\;{time}_{i},\;D_{i}\right)$ to $h_{2}^{list}$.
3. Private-key-extract query.
  If coin=0, then C stops the session. Otherwise, C chooses a random number $d_{1i}\in Z_{q}^{*}$ as a private key of ${PID}_{i}$, and generates another two random numbers $d_{2i}{,a}_{i}\in Z_{q}^{*}$ , and C sets $P_{1i}=d_{1i}\cdot P$, $h_{1i}\leftarrow a_{i}$ and $P_{2i}\leftarrow d_{2i}\cdot P-h_{1i}\cdot P_{PKG}$. C adds $\left({PID}_{i},\;d_{1i},\;P_{1i}\right)$ and $\left({PID}_{i},\;d_{2i},\;P_{2i}\right)$ to $d_{1}^{list}$ and $d_{2}^{list}$ respectively, then C returns $d_{1i}$ to A as a response.
4. Partial-private-key-extract query.
  If coin=0, then C stops the session. Otherwise, C looks up $d_{2}^{list}$ and checks whether the tuple $\left({PID}_{i},\;d_{2i},\;P_{2i}\right)$ exist in the list first. If yes, C returns $d_{2i}$ to A as a response. Else, C makes a private-key-extract query on ${PID}_{i}$ itself and returns $d_{2i}$ to A as a response.
5. Sign query.
  A makes a query on ${PID}_{i}$ and $M_{i}$. C looks up $\left({PID}_{i},\;P_{1i},\;P_{2i},\;T_{i},\;B_{i},\;coin\right)$ firstly. If coin=0, then C finds $\left({PID}_{i},\;d_{1i},\;P_{1i}\right)$ and $\left({PID}_{i},\;d_{2i},\;P_{2i}\right)$ in $d_{1}^{list}$ and $d_{2}^{list}$ respectively, and generates two random numbers $b_{i}{,v}_{i}\in Z_{q}^{*}$, and sets $h_{2i}\leftarrow b_{i}$, $L_{i}=v_{i}\cdot P-P_{2i}-h_{1i}\cdot P_{PKG}-P_{1i}\cdot b_{i}$. C returns $\left({PID}_{i},\;M_{i},\;v_{i},\;L_{i},\;P_{1i},\;P_{2i}\right)$ to A as a response. Note that it is easy to verify the equation $v_{i}\cdot P=L_{i}+P_{2i}+c\cdot P_{PKG}+P_{1i}\cdot h_{2i}$ holds.
  If coin=1, the signature is ordinary because that C knows the private key and partial private key.
6. Finally, A outputs $\left({PID}^{*},\;M^{*},\;v^{*}\right)$. Note that $\left({PID}^{*},\;M^{*}\right)$ is not submitted to the query of private key, partial private key and signature. If coin=1, then C stops the simulation. Otherwise, according to [32], A can generate another valid signature with the same random tape but the different value of $h_{1i}$ as follows:
  (13) $$v^{\prime }\cdot P=L_{i}+P_{2i}+h_{1i}^{\prime }\cdot P_{PKG}+P_{1i}\cdot h_{2i}$$
  (14) $$v^{\prime \prime }\cdot P=L_{i}+P_{2i}+h_{1i}^{\prime \prime }\cdot P_{PKG}+P_{1i}\cdot h_{2i}$$

According to the Equations (13) and (14), we can get:

(15) $$v^{\prime }-v^{\prime \prime }\cdot P=(h_{1i}^{\prime }-h_{1i}^{\prime \prime })x\cdot P$$

(16) $$x=(v^{\prime }-v^{\prime \prime })/(h_{1i}^{\prime }-h_{1i}^{\prime \prime })\mathrm{mod}\;q$$

Thus, C outputs x as the solution of ECDLP problem $P_{PKG}=x\cdot P$. It is contradict to solve the ECDLP hard problem.  ☐

**Theorem** **2.** *Our scheme is secure against adaptive chosen message attacks of the super adversary A2 under the random oracle*.

**Proof.**  There are two roles in the game, the challenger C and the adversary A. C use A as a subroutine to break our scheme via solving the ECDLP problem with a non-negligible probability. C picks a random number $s\in Z_{q}^{*}$ as the master key of the PKG and sets $P_{PKG}=s\cdot P$, then C generates the system $\mathrm{params}\;\left({p,\;q,\;P,\;P}_{PKG},\;h_{1},\;h_{2}\right)$. C sends s and the $\mathrm{params}\;\left(p,\;q,\;P,\;P_{PKG},\;h_{1},\;h_{2}\right)$ to the adversary A2. C maintains 4 lists $h_{1}^{list},\;h_{2}^{list},\;d_{1}^{list},\;d_{2}^{list},$ which are initially empty. C answers $h_{1}$ query and $h_{2}$ query like it does in the first oracle query phase. C simulates another oracles queried by A as follows.

1. Partial-private-key-extract query. If coin=0, then C looks up $h_{1}^{list}$ and identifies the tuple $\left({PID}_{i},\;P_{1i},\;P_{2i},\;T_{i},\;B_{i},\;coin\right)$ , then C picks a random number $k_{i}\in Z_{q}^{*}$, and calculates $d_{2i}=k_{i}+s\times h_{1i}\mathrm{mod}\;q$. C adds $\left({PID}_{i},⊥,\;P_{1i}\right)$ and $\left({PID}_{i},\;d_{2i},\;P_{2i}\right)$ to $d_{1}^{list}$ and $d_{2}^{list}$ respectively. C returns $d_{2i}$ to A as a response.
  If coin=1, then C looks up $h_{1}^{list}$ and identifies the tuple $\left({PID}_{i},\;P_{1i},\;P_{2i},\;T_{i},\;B_{i},\;coin\right)$, then C picks two random numbers $a_{i},\;k_{i}\in Z_{q}^{*}$. C sets $d_{1i}\leftarrow a_{i}$, and calculates $d_{2i}=k_{i}+s\times h_{1i}\;\mathrm{mod}\;q$ and $P_{1i}=d_{1i}\cdot P$. C adds $\left({PID}_{i},\;d_{1i},\;P_{1i}\right)$ and $\left({PID}_{i},\;d_{2i},\;P_{2i}\right)$ to $d_{1}^{list}$ and $d_{2}^{list}$ respectively. C returns $d_{2i}$ to A as a response.
2. Private-key-extract query. When A makes the query, C does as follows:
  If coin=0, then C stops the session. Otherwise, C looks up $d_{1}^{list}$ and identifies the tuple $\left({PID}_{i},\;d_{1i},\;P_{1i}\right)$, and sends $d_{1i}$ to A as a response. If there is no tuple in the list, C makes a partial-private-key-extract query on ${PID}_{i}$ itself, then C returns $d_{1i}$ as a response.
3. Sign query. A makes a query on ${PID}_{i}$ and $M_{i}$. C looks up $\left({PID}_{i},\;P_{1i},\;P_{2i},\;T_{i},\;B_{i},\;coin\right)$ firstly. If coin=0, then C finds $\left({PID}_{i},⊥\;,P_{1i}\right)$ and $\left({PID}_{i},d_{2i},P_{2i}\right)$ in $d_{1}^{list}$ and $d_{2}^{list}$ respectively. C picks three random numbers $x,b_{i},v_{i}\in Z_{q}^{*}$ and sets $P_{1i}=x\cdot P$, $h_{2i}\leftarrow b_{i}$ and $L_{i}=v_{i}\cdot P-P_{2i}-h_{1i}\cdot P_{PKG}-P_{1i}\cdot b_{i}$. C returns $\left({PID}_{i},M_{i},v_{i},L_{i},P_{1i},P_{2i}\right)$ to A as a response. If coin=1, the signature is ordinary.
4. Finally, A outputs $\left({PID}^{*},M^{*},v^{*}\right)$. Note that $\left({PID}^{*},\;M^{*}\right)$ is not submitted to the query of private key and signature. If coin=1, then C stops the simulation. Otherwise, according to [32], A can generate another valid signature with the same random tape but the different value of $b_{i}$ as follows:
  (17) $$v^{\prime }\cdot P=L_{i}+P_{2i}+h_{1i}\cdot P_{PKG}+P_{1i}\cdot b_{i}^{\prime }$$
  (18) $$v^{\prime \prime }\cdot P=L_{i}+P_{2i}+h_{1i}\cdot P_{PKG}+P_{1i}\cdot b_{i}^{\prime \prime }$$

According to the Equations (17) and (18), we can obtain:

(19) $$v^{\prime }-v^{\prime \prime }\cdot P=(b_{1i}^{\prime }-b_{1i}^{\prime \prime })x\cdot P$$

(20) $$x=(v^{\prime }-v^{\prime \prime })/(b_{1i}^{\prime }-b_{1i}^{\prime \prime })\mathrm{mod}\;q$$

Thus, C outputs *x* as the solution ECDLP problem $P_{1i}=x\cdot P$.  ☐

**Theorem** **3.** *Our scheme is secure against the super adversary A3 attacks*.

**Proof.**  In this scenario, A3 presents a malicious PKG who can obtain the master key s of the PKG and forge the secret key $d_{i}^{\prime }$ at will. His target is to obtain the successful verification by another valid VSN entities. Nevertheless, a valid signature cannot be produced without the unique secret key $d_{1}$. In our scheme, PID is generated via calculating ${PID}_{i}=r\times h_{1}\left(H_{1}\left|\left|P_{1}\right|\right|T\right)+n_{i}+d_{1}\mathrm{mod}\;q$. Thus, the adversary has to obtain $d_{1}$ from valid users. It is difficult to steal $d_{1}$ from the smart card without the user’s PW because that there is no $d_{1}$ stored in the smart card after logging out. Moreover, because of the intractability of ECDLP problem, the adversary cannot obtain $d_{1}$ from $P_{1}=d_{1}\cdot P$ and the TA’s master key r from $P_{TA}=r\cdot P$. The probability of this malicious PKG managing to collude with the TA and stealing the master key from the TA is negligible. Therefore, the scheme is secure against this kind of adversary attacks, which leaves the opportunity to adversaries in [26,27], though.  ☐

### 5.2. Experiment 2

In the register phrase, the proposed scheme can resist against the inner attacker from the TA. Every pseudo identity ${PID}_{i}$ contains the TA’s master secret key r and the user’s private key $d_{1}$. Without knowing the user’s private key $d_{1}$, any insider adversaries fail to impersonate the valid user to proceed with the next step. In this experiment, if the adversary cannot forge a valid pseudo identity ${PID}_{i}$ verified by PKG successfully, the proposed scheme is secure against impersonation attacks by insider adversaries. The secure module with proof in the random oracle is as follows:

**Proof.**  Suppose there is an adversary A that represents an inner attacker from TA and he is able to access TA’s master secret key r but cannot get user’s private key $d_{1}$ or forge it. This assumption is reasonable, because that the adversary has no right to modify the ID table in the TA. We construct a challenger C, which can solve ECDLP with a non-negligible probability by running A as a subroutine. C picks ${ID}^{*}$ as a challenged identity and sets system public key $P_{TA}=r\cdot P$, in which $r\in Z_{q}^{*}$ is the master secret key, then C sends the system $\mathrm{params}\;\left(p,\;q,\;P,\;P_{TA},\;h\right)$ to the adversary A. C maintains 3 lists $h^{list},d_{1}^{list}$ and ${TA}^{list}$ which are initially empty.

1. h query. C maintains a list with the form of $\left({ID}_{i},\;P_{1i},\;T_{i},\;H_{1},\delta_{i},\;coin\right)$. When A makes a query on $\left({ID}_{i},\;P_{1i},\;T_{i},\;H_{1}\right)$, C checks whether the tuple exist in the list $h^{list}$. If so, C responds $\delta_{i}=h\left({ID}_{i},\;P_{1i},\;T_{i},\;H_{1}\right)$; otherwise, C generates a random number $coin\leftarrow_{{\;}_{R}}\left\{0,\;1\right\}$ and sets Pr[coin=0]=η, in which coin=0 means that this ${ID}_{i}$ is the challenged identity. Then C picks $\delta_{i}\leftarrow_{{\;}_{R}}Z_{q}^{*}$ and sends $\delta_{i}=h\left({ID}_{i},\;P_{1i},\;T_{i},\;H_{1}\right)$ to A as a response. C adds $\left({ID}_{i},\;P_{1i},\;T_{i},\;H_{1},\;\delta_{i},\;coin\right)$ to $h^{list}$.
2. Master-secret-key query. When A makes the query, C does as follows:
  C looks up $\left({ID}_{i},\;P_{1i},\;T_{i},\;H_{1},\;\delta_{i},\;coin\right)$ firstly. If coin=1, C picks a random number $a_{i}\in Z_{q}^{*}$. C sets $d_{1i}\leftarrow a_{i}$ and calculates $P_{1i}=d_{1i}\cdot P$, then C adds $\left({ID}_{i},{\;d}_{1i},\;P_{1i}\right)$ and $\left({ID}_{i},\;r\right)$ to $d_{1}^{list}$ and ${TA}^{list}$ respectively. C returns r to A as a response.
  If coin=0, C adds $\left({ID}_{i},\;⊥,\;P_{1i}\right)$ and $\left({ID}_{i},\;r\right)$ to $d_{1}^{list}$ and ${TA}^{list}$ respectively. C returns r to A as a response.
3. Private-key-extract query. C looks up $\left({ID}_{i},\;P_{1i},\;T_{i},\;H_{1},\;\delta_{i},\;coin\right)$ firstly. If coin=0, then C stops the session. Otherwise, C looks up $d_{1}^{list}$ and identifies the tuple $\left({PID}_{i},{\;d}_{1i},\;P_{1i}\right)$. Then C sends $d_{1i}$ to A as a response. If there is no tuple in the list, C makes a master-secret-key query on ${ID}_{i}$ itself, then C returns $d_{1i}$ as a response.
4. PID query. A makes a ${PID}_{i}$ query on ${ID}_{i}$. C looks up $\left({ID}_{i},\;P_{1i},\;T_{i},\;H_{1},\;\delta_{i},\;coin\right)$ firstly. If coin=0, then C finds $\left({ID}_{i},\;⊥,\;P_{1i}\right)$ and $\left({ID}_{i},\;r\right)$ in $d_{1}^{list}$ and ${TA}^{list}$ respectively. C picks three random numbers $x,b_{i},{PID}_{i}\in Z_{q}^{*}$, then C sets $P_{1i}=x\cdot P$, $h_{i}\leftarrow b_{i}$ and $N_{i}={PID}_{i}\cdot P-b_{i}\cdot P_{TA}-P_{1i}$. C returns $\left({ID}_{i},\;v_{i},\;N_{i},\;P_{1i}\right)$ to A as a response. If coin=1, the ${PID}_{i}$ is ordinary.
5. Finally, A outputs $\left({ID}^{*},\;{PID}^{*}\right)$. Note that $\left({ID}^{*},\;{PID}^{*}\right)$ is not submitted to the query of private key and PID. If coin=1, then C stops the simulation. Otherwise, according to [32], A can generate another valid pseudo identity with the same random tape but the different coefficient *m* of $P_{1i}$ as follows:
  (21) $${PID}^{\prime }\cdot P=N_{i}+P_{1i}+P_{TA}\cdot b_{i}$$
  (22) $${PID}^{\prime \prime }\cdot P=N_{i}+m\cdot P_{1i}+P_{TA}\cdot b_{i}$$

According to the Equations (21) and (22), we can obtain:

(23) $$\left({PID}^{\prime }-{PID}^{\prime \prime }\right)\cdot P=\left(1-m\right)x\cdot P$$

(24) $$x=\left({PID}^{\prime }-{PID}^{\prime \prime }\right)/\left(1-m\right)\mathrm{mod}\;q$$

Thus, C outputs x as the solution ECDLP problem $P_{1i}=x\cdot P$. The ability of solving the ECDLP problem contradicts the hardness of the ECDLP problem. Therefore, the proposed scheme is secure against impersonation attacks by insider attackers from TA.  ☐

### 5.3. Experiment 3

**Figure 6:**
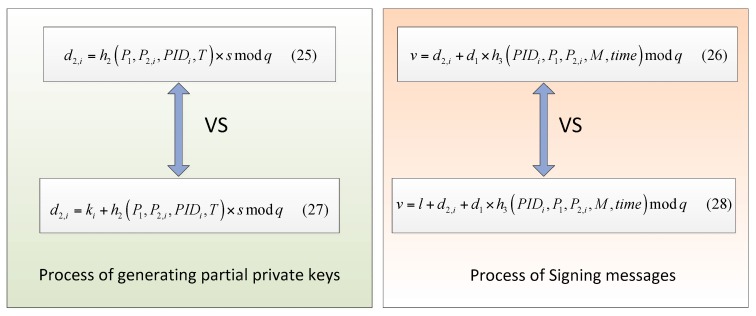
Comparison of two different schemes.

In the authentication process, we make use of two elements to provide the freshness of the signed message. The comparison of different schemes in the Figure 6 shows the importance of $k_{i}$ and l in the signed message $\left\{{PID}_{i},\;P_{1},P_{2,i},\;M,\;L,\;T,\;v,\;time\right\}$.

**Proof.**  Note that without $k_{i}$ and l it is easy for adversaries to get master secret key s and of PKG and private key $d_{1}$ in the Equations (25) and (26).

The adversary can acquire $\left\{PID,\;P_{2},\;d_{2}\right\}$ from the public channel. It is easy to compute s by following steps:

(1) Get $P_{1}$ and T from the public message $\left\{PID,\;H_{1},\;P_{1},\;N,\;T\right\}$.
(2) Get $\left\{PID,\;P_{2},\;d_{2}\right\}$ from the public channel.
(3) Compute s:
  (29) $$d_{2,i}=h_{2}\left(P_{1},\;P_{2,i},\;{PID}_{i},\;T\right)\times s\;\mathrm{mod}\;q$$
  (30) $$s={\;d}_{2,i}/h_{2}\left(P_{1},\;P_{2,i},\;{PID}_{i},\;T\right)\;\mathrm{mod}\;q$$

It is easy to compute $d_{1}$ for adversaries in the same way.

(1) Get $d_{2}$ from the public message $\left\{PID,\;P_{2},\;d_{2}\right\}$.
(2) Compute $h_{3}\left({PID}_{i},P_{1},\;P_{2,i},\;M,\;time\right)$ by $\left\{{PID}_{i},\;P_{1},\;P_{2,i},\;M,\;T,\;v,\;time\right\}$ from the public channel.
(3) Compute $d_{1}$:
  (31) $$v=d_{2,i}+d_{1}^{\;}\times h_{3}\left({PID}_{i},P_{1}{,P}_{2,i},M,time\right)\;\mathrm{mod}\;q$$
  (32) $$d_{1}^{\;}=\left(v-d_{2,i}\right)/h_{3}\left({PID}_{i},P_{1}{,P}_{2,i},M,time\right)\;\mathrm{mod}\;q$$
   ☐

In order to protect the master key of PKG and user’s private key, we add two elements to the Equations (25) and (26). The secure module with proof using random oracle is as follows:

In this experiment, assume that to forge the valid k that make $d_{2,i}=k_{i}+h_{2}\left(P_{1},P_{2,i},{PID}_{i},T\right)\times s\;\mathrm{mod}\;q,\;\left(i=1\ldots m\right)$ be verified successfully is the adversary’s target. That means the adversary can compute right k and then achieve the value of s.

**Proof.**  Suppose there is an adversary A that is not able to access the master key of the PKG or the secret value k but can access the partial private key $d_{2}$ of users. Note that in this experiment the adversary just play this game by himself to forge the k, so $d_{2}$ can be seemed as a public number without being verified by others. We construct a challenger C, which can solve ECDLP with a non-negligible probability by running A as a subroutine. C picks ${PID}^{*}$ as a challenged identity and sets system public key $P_{PKG}=s\cdot P$, in which $s\in Z_{q}^{*}$ is the master secret key, then C sends the system $\mathrm{params}\left(p,q,P,P_{PKG},h\right)$ to the adversary A. C maintains 2 lists $h^{list}$ and ${PKG}^{list}$ which are initially empty.

1. h query. C maintains a list with the form of $\left({PID}_{i},P_{1i},P_{2i},\theta_{i},coin\right)$. When A makes a query on $\left({PID}_{i},P_{1i},P_{2i}\right)$, C checks whether the tuple exist in the list $h^{list}$. If so, C responds $\theta_{i}=h\left({PID}_{i},P_{1i},P_{2i}\right)$; otherwise, C generates a random number $coin\leftarrow_{{\;}_{R}}\left\{0,1\right\}$ and sets Pr[coin=0]=η, in which coin=0 means that this ${PID}_{i}$ is the challenged identity. Then C picks ${\theta_{i}}_{i}\leftarrow_{{\;}_{R}}Z_{q}^{*}$ and sends $\theta_{i}=h\left({PID}_{i},P_{1i},P_{2i}\right)$ to A as a response. C adds $\left({PID}_{i},P_{1i},P_{2i},\theta_{i},coin\right)$ to $h^{list}$.
2. Master-secret-key query. When A makes the query, C does as follows:
  C looks up $\left({PID}_{i},P_{1i},P_{2i},\theta_{i},coin\right)$ firstly. If coin=1, C adds $\left({PID}_{i},s\right)$ to ${PKG}^{list}$. C returns s to A as a response.
  If coin=0. , then C stops the session.
3. k query. When A makes a k query on ${PID}_{i}$. C looks up $\left({PID}_{i},P_{1i},P_{2i},\theta_{i},coin\right)$ firstly. If coin=0, then C finds $\left({PID}_{i},s\right)$ in the ${PKG}^{list}$. C picks a random number $b_{i}\in Z_{q}^{*}$, then C sets $h_{i}\leftarrow b_{i}$ and $D_{i}=k_{i}\cdot P+b_{i}\cdot P_{PKG}$, in which $D_{i}=d_{2,i}\cdot P$. C returns $\left({PID}_{i},k_{i},D_{i}\right)$ to A as a response. If coin=1 , the $k_{i}$ is ordinary.
4. Finally, A outputs $\left({PID}^{*},k^{*}\right)$. Note that $\left({PID}^{*},k^{*}\right)$ is not submitted to the query of *k*. If coin=1, then C stops the simulation. Otherwise, according to [32], A can generate another valid pseudo identity with the same random tape but the different values of $b_{i}$ as follows:
  (33) $$k^{\prime }\cdot P=D_{i}-P_{PKG}\cdot b_{i}^{\prime }$$
  (34) $$k^{\prime \prime }\cdot P=D_{i}-P_{PKG}\cdot b_{i}^{\prime \prime }$$

According to the Equations (33) and (34), we can obtain

(35) $$\left(k^{\prime }-k^{\prime \prime }\right)\cdot P=\left(b_{i}^{\prime \prime }-b_{i}^{\prime }\right)s\cdot P$$

(36) $$s=\left(k^{\prime }-k^{\prime \prime }\right)/\left(b_{i}^{\prime \prime }-b_{i}^{\prime }\right)\mathrm{mod}\;q$$

Thus, C outputs s as the solution ECDLP problem $P_{PKG}=s\cdot P$. The ability of solving the ECDLP problem contradicts the hardness of the ECDLP problem. Thus, the adversary cannot forge a valid k to compute the master key of the PKG.

The freshness of *L* in the Equation (27) that has the same function with *k* is to protect the private key of users. We will omit the same proof.  ☐

### 5.4. Experiment 4

The proposed scheme implements a location-based method, with which every RSU can acquire their current coordinates and apply them in every signature. The freshness of current location protects RSUs from being captured and compromised.

**Figure 7:**
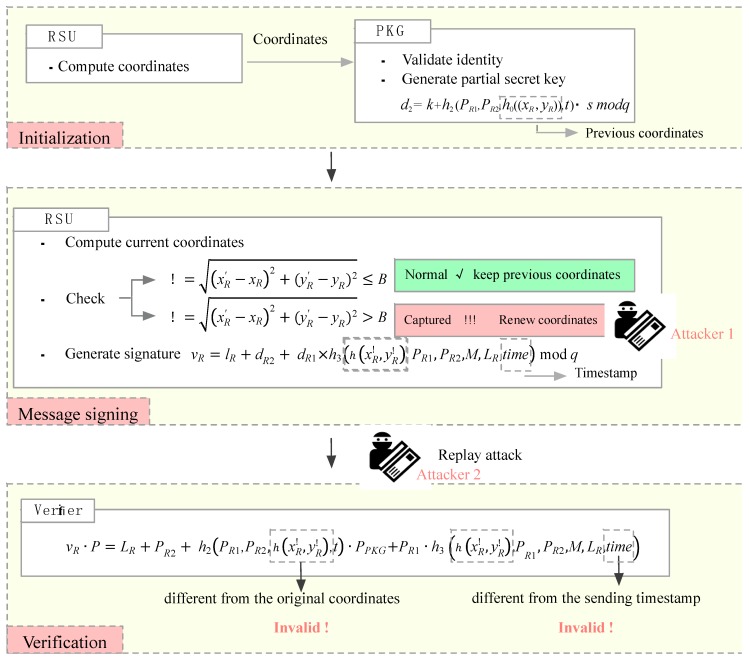
Freshness of timestamp and coordinates.

Furthermore, every signature including a timestamp time is to record the current sending time of the signer. Verifiers can check out the replay attack easily by validating the freshness of receiving ${time}^{*}$. If ${time}^{*}-time\text{>}\Delta T$, in which ΔT indicates the valid time interval, the verifier will reject the signature. Figure 7 shows the function of the coordinates $\left(x_{R}^{\prime },y_{R}^{\prime }\right)$ and the timestamp ${time}^{*}$ included in the signature.

**Analysis:** In Figure 7, there are two attackers. The first one implements node captured attacks and the second one captures valid signatures to carry out replay attacks. Because of the different location, the attacker 1 can access any of information in the compromised RSU expect $d_{2}$. The ability of this kind of attackers is weaker than the adversary A3 as mentioned in the experiment 1. The ability of the attacker 2 is as same as the adversary A1 that is not able to access the master key of the PKG or the secret keys of users. However, they all fail to generate valid signatures and the proof is mentioned above.

## 6. Security Analysis

Considering the implementation costs, it’s difficult to make all communication channels secure in VSNs. In our scheme, all communication channels are public, which is different from that in [27]. The TA is credible without being stolen its secret key by adversaries and its master key must be strongly protected by hardware technology.

The proposed scheme is on the basis of the CLPKC. Thus, our scheme can provide message authentication and integrity. The unforgeability against adaptive chosen messages attacks is defined in Section 5, which also provides the details of the scheme and its security proof. Thus, our scheme supports message authentication, integrity and unforgeability. The other security analyses are given in details as follows.

### 6.1. Traceability

The proposed scheme provides traceability. If one message is disputable, TA, the only authorized entity, can perform the tracing procedure and extract the real identity from the signature $\left\{PID,P_{1},P_{2},M,\;L,\;T,\;v,\;time\right\}$ via calculating $PID\cdot P=P_{TA}\cdot h_{1}\left(H_{1}||P_{1}||T\right)+N+P_{1}$, in which $H_{1}$ and N are stored in its repository. If one $H_{1,j}$ satisfied the equation as above, the TA can obtain the ${\left(ID_{j}\right)}_{P_{TA}}$ from ${\left({ID}_{j}\right)}_{P_{TA}}⊕h_{0}\left({PW}_{j}⊕b\right)=H_{1,j}$ and extract the real identity ${ID}_{j}$ by decrypting ${\left(ID_{j}\right)}_{P_{TA}}$ using the secret key r of the TA. Note that no one can obtain ${ID}_{j}$ since r is only known by the TA itself.

### 6.2. Unlinkability

Unlinkability is that an adversary cannot link the signature messages generated by the same vehicle. Every signature message $\left\{PID,\;P_{1},P_{2},M,\;L,\;T,\;v,\;time\right\}$ is different, because it is signed by different PIDs and related partial private keys. $PID=r\times h_{1}\left(H_{1}||P_{1}||T\right)+n+d_{1}\;\mathrm{mod}\;q$ is generated by the random number n which any adversary who want to obtain will encounter the ECDLP problem. Therefore, the proposed scheme supports unlinkability.

### 6.3. Resistance against Impersonation Attacks

An adversary can impersonate a legitimate user to access RSUs by generating a valid PID and a signature message $\left\{PID,\;P_{1},P_{2},\;M,\;L,\;T,\;v,\;time\right\}$. With our scheme, every pseudo identity ${PID}_{i}$ contains the TA’s master secret key r and the user’s private key $d_{1}$. Furthermore, every signature includes the PKG’s master secret key s and $d_{1}$. Without knowing the user’s private key $d_{1}$, any insider adversaries of the PKG fail to calculate the valid PIDs and signatures. The proof is given in Section 5.2. Note that $d_{1}$ is not transferred through any channels or stored in the smart card, and when the user does not input his valid PW, the smart card cannot obtain the valid $d_{1}$. Therefore, it is difficult for any adversaries to obtain $d_{1}$ by various methods of attack and because of the ECDLP problems, they cannot extract $d_{1}$ from $P_{1}=d_{1}\cdot P$. Assume that there is an adversary who eavesdrops the information $\left\{PID_{1},\;H_{1},N,T\right\}$ of one user or eavesdrops $\left\{P_{2},d_{2}\right\}$ from the PKG through the public channels instead of the valid user, they all fail to generate valid PIDs and signatures because of lacking $d_{1}$.

### 6.4. Resistance against Node Compromise Attacks and Node Replication Attacks

The proposed scheme can prevent against node compromise and replication attacks to a large extent, and it incorporates three subsections according to the attacker’s abilities:

(1) We assume that an adversary captures a node ${RSU}_{i}$ and does not move this node to another location. The adversary extracts all stored information from the node, however, the information is independent of other nodes. And the adversary modifies the safety messages according to his specific needs and causes data anomalies. The position-based authentication method can help the PKG identify the malicious node based on its coordinates. Note that the adversary cannot change the node’s coordinates or it will fail to be verified. In addition, there is no need to compromise the anchor node because this type of node does not contain important traffic information or privacy of users.
(2) Assuming that an adversary captures a node ${RSU}_{i}$ and replicates it in another place, this new replicated node executes the same program as before. However, the node cannot generate valid signatures because it computes a current position ${ID}_{R2}^{\prime }=h_{0}\left(x_{R}^{\prime },\;y_{R}^{\prime }\right)$ according to new nearby anchor nodes. Note that ${ID}_{R2}^{\prime }$ is different from the original ${ID}_{R2}$ in $d_{R2}=k_{R}+h_{2}\left(P_{R1},\;P_{R2},\;{ID}_{R2},t\right)\times s\;\mathrm{mod}\;q$. Therefore, these malicious nodes will be identified quickly by the verifiers because of their invalid signatures.
(3) We assume that there is a powerful adversary who can modify the original program in the node after capturing and replicating it in another location. Note that the adversary cannot change ${ID}_{R2}$ in $d_{R2}=k_{R}+h_{2}\left(P_{R1},\;P_{R2},\;{ID}_{R2},\;t\right)\times s\;\mathrm{mod}\;q$. without knowing the master private key s. Therefore, to generate a valid signature the adversary only uses the original value of ${ID}_{R2}$ instead of updating it vie the new anchor nodes. Unfortunately, these malicious nodes will be identified rapidly by the detection mechanism of the proposed method because of their wrong coordinates. When the adjacent anchor nodes receive the signature $\left\{\left(x_{R}^{\prime },\;y_{R}^{\prime }\right),\;{ID}_{R2}^{\prime },\;P_{R1},\;P_{R2},\;M,\;L_{R},\;t,\;time,\;v_{R}\right\}$, they compare their current location calculated by $\left(x_{R}^{\prime },\;y_{R}^{\prime }\right)$ with the previous one, which is obtained from the GPS. If the value significantly changes, then abnormal RSUs must be surrounding the anchor node, and the anchor node will generate an alert to the PKG. Therefore, our scheme can withstand node compromise and replication attacks.

### 6.5. Resistance against Stolen Smart Card Attacks

We assume that the smart card of user $U_{i}$ has been lost or stolen by an adversary. The adversary can then extract the parameters $\left\{h_{0}\left(PW⊕b\right),h_{0}\left(ID\right),s_{2},P_{1},P_{2},d_{2},\;b,\;T,\;N,H_{1}\right\}$ stored in the smart card, although the user’s independent information $\left\{d_{1},\;PW,\;ID,s_{1}\right\}$ is not contained in the card. Moreover, calculating or guessing the user’s correct value of ${PW}_{i}$, ${ID}_{i}$ and $d_{1,i}$ is difficult. Therefore, the adversary cannot acquire the secret credentials of the target user. In addition, our proposal does not maintain any real-identity table, such as the RSU’s ${ID}_{R1},{ID}_{R2}$ in the PKG and the user’s ${ID}_{i}$ in the TA to safeguard against stolen identity attacks by privileged insiders.

### 6.6. Resistance against Replay Attacks

All valid signatures maintain the timestamp time. The verifiers can find the replay message via checking whether ${time}^{*}-time\leq \Delta T$. Therefore, the proposed scheme can withstand the replay attacks. Table 2 shows the security compared with recently proposed authentication schemes in [15,22,27].

## 7. Performance Evaluation

In this section, we analyze the computational costs and transmission overhead of our scheme. We implement our scheme using a Lenovo computer (Beijing, China) equipped with an Intel I7 dual-core processor, a 2.60 GHZ clock frequency and 1 gigabytes of memory running the VMWare Ubuntu12.03 operating system. For our ID-based scheme with ECC, we use an additive group G generated by a point p with the order q on the secp256r1 elliptic curve to achieve the security level of 128 bits, in which p and q are two 256-bit prime numbers. For the bilinear pairings based scheme, we use the bilinear pairings $y=x^{3}+b\;\mathrm{mod}\;q$ with embedding degree 12 and the q is a 256-bit prime number.

### 7.1. Computational Overhead

For convenience, we define some notations about the execution time as follows. First, Let $T_{bp}$ denote the execution time of a bilinear pairing operation, $T_{hmtp}$ be the time to execute one MapToPoint hash operation that is different from the general hash function operation $T_{h}$. Then $T_{epm}$ and $T_{epa}$ denote the time of executing one point multiplication and one point addition over an elliptic curve respectively. $T_{RSSI}$ represents the time of computing coordinates of a RSU. At last, $T_{ecc-sign}$ and $T_{ecc-verify}$ represent the time of signing one message and verifying one message based on the secp256r1 elliptic curve respectively. The execution time of aforementioned operations is listed in Table 3.

We compare the execution time of our scheme with other related works in [15,19,22,27]. Table 4 shows the execution time of signing a single message and a batch verification of five different schemes.

In our scheme, a vehicle signing a message takes 2.3 μs and the RSU processing 13.4 μs, which is slightly slower than that of Lo’s scheme. However, the proposed scheme provides better scalability without providing a specific secure channel, which is different from Lo’s scheme, and our scheme can resist node compromise attacks, which other schemes do not consider. Therefore, the proposed scheme is efficient in terms of computational overhead and more secure than other schemes. More precisely, the proposed scheme can obtain better trade-offs than the four other schemes.

Next, we compare the performance of batch verification in the proposed scheme with that of the other three proposed ID-based batch verification schemes.

**Figure 8:**
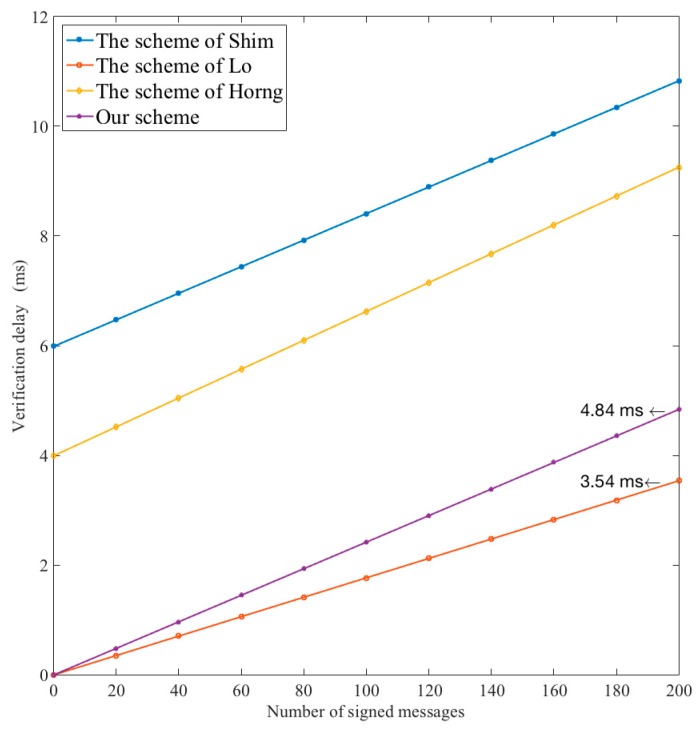
Comparison of execution time for the batch verification.

Figure 8 shows the relationship between the density of signing messages at a VSN entity inside its wireless range and the verification delay. The verification delay of the proposed scheme, which is 6.5 μs for one message, is slightly longer than the one in Lo’s scheme. However, the difference is small, and the safety of our scheme is enhanced largely.

### 7.2. Communication Overhead 

In this subsection, we analyze the communication overhead in our scheme and compare it with other proposed schemes. In our scheme, the signed message contains $\left\{PID,\;P_{1},P_{2},\;M,\;L,\;T,\;v,\;time\right\}$ and $\left\{\left(x_{R}^{\prime },y_{R}^{\prime }\right),\;{ID}_{R2}^{\prime },\;P_{R1},\;P_{R2},\;M,\;L_{R},\;t,\;time,\;v_{R}\right\}$ for a vehicle and a RSU respectively. Since the length of p and q is 256 bits, so the length of element of G is 512 bits. The length of M is about 256 bits, which is the same as the value of the general hash function. Let timestamp, expiration time and the coordinates of one node be 32 bits. Table 5 shows the communication costs of our scheme and Table 6 shows the comparison of communication overhead among four schemes.

The communication overhead of proposed scheme is about 296 bytes and 300 bytes for a vehicle and a RSU respectively. To reduce the communication overhead, the key point in the proposed scheme is how to reduce the costs of the elements in G. Shim [22] developed a method, which can reduce the size of a point (x,y) in G. In this method, the entity (RSU or vehicle) only sends the x-coordinate of the point, and the receiver can acquire the y-coordinate by calculating the square root. Therefore, the size of the (x,y) is reduced by applying this method, and in our scheme, the total communication overhead for a vehicle is about 256 + 256 + 256 + 256 + 256 + 256 + 32 + 32 = 1600 bits = 200 bytes, and for a RSU is about 32 + 256 + 256 + 256 + 256 + 256 + 256 + 32 + 32 = 1632 bits = 204 bytes. Therefore, the proposed method obtains the smallest communication overhead compared with the other three schemes.

**Figure 9:**
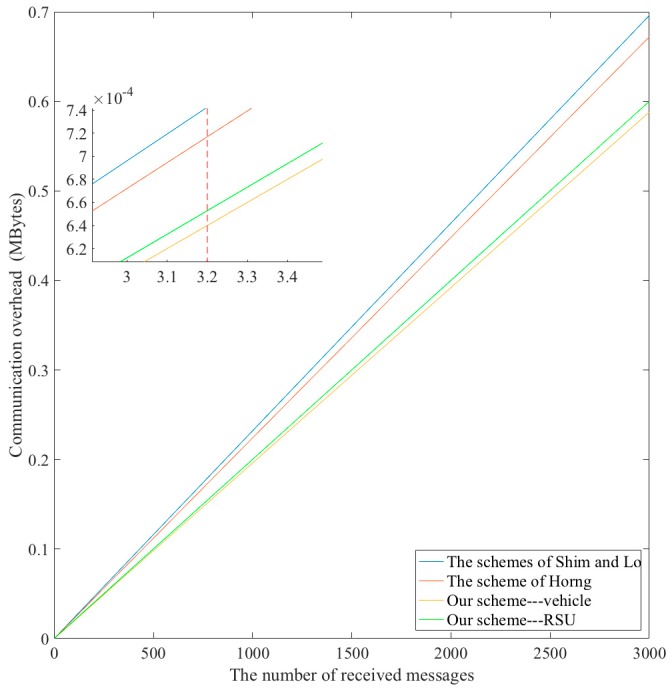
Comparison of the communication overhead.

Figure 9 shows the relationship between the communication overhead and the number of received messages. Obviously, the communication costs for RSUs are the smallest for the proposed scheme compared with the other three schemes.

In summary, the proposed scheme requires a smaller communication bandwidth than the other schemes when it transmits signed messages to other VSN entities.

## 8. Conclusions

In this work, we have proposed an enhanced secure ID-based, certificateless authentication scheme for VSNs that supports batch verification and conditional privacy-preserving authentication. In addition, the proposed scheme provides compromised-RSU detection and an alarm mechanism, which many related works have not considered. The security analysis shows that the proposed scheme is secure against adaptive chosen message attacks by three types of adversaries under a random oracle. Furthermore, the proposed scheme can resist against major threats like impersonation attacks, node replication attacks, hardware (RSU) tampering attacks, stolen smart card attacks and replay attacks. At last, the scheme can obtain better trade-offs between security and efficiency than other proposed schemes.

In future studies, researchers will focus on different network architectures of VSNs. We will focus on different scenarios in VSNs and consider compatible secure models that can co-exist in heterogeneous networks of VSNs. A designed scheme with better compatibility and scalability will be more suitable for the VSNs.

## Figures and Tables

**Table 1:** List of notations.

Symbol	Descriptions	Symbol	Descriptions
RSU	A roadside unit	$d_{1}$	A secret key of a user
TA	A Trusted Authority	$d_{2}$	The partial secret keys of a user issued by the PKG
PKG	A Private Key Generator	$P_{1}$	A public key of a user
n	A k-bit prime number	$P_{2}$	A public key of users issued by the PKG
$F_{n}$	A finite field with n elements	r	A private key of the TA
$E\left(F_{n}\right)$	An Elliptic Curve over a finite field $F_{n}$, $y^{2}=x^{3}+ax+bmodn$, $a,\;b,\;x,\;y\in F_{n}$	s	A private key of the PKG
b	A secret number in a smart card	PW	The password of the smart card
G	An additive group with the order *q*	$P_{TA}$	A public key of the TA
q	The order of the group *G*	$P_{PKG}$	A public key of the PKG
P	The point generator of the group *G_q_*	time	A timestamp
PID	The pseudo identity of a user	⊕	Exclusive-OR operation
RID	The real identity of a user	∥	Message concatenation operation

**Table 2:** Security Comparisons of Related Schemes and Our Scheme.

The Types of Attacks	Calandriello ’s Scheme	Shim’s Scheme	Lo’s Scheme	Our Scheme
Traceability	No	YES	YES	YES
Unlinkability	YES	YES	YES	YES
Resistance to impersonation attack	YES	YES	YES	YES
Resistance to node replication attack	No	No	No	YES
Resistance to node compromise attack	No	No	No	YES
Resistance against replay attack	No	YES	YES	YES

**Table 3:** Execution Time of Different Operations.

Operation	Execution Time (Microsecond)
$T_{bp}$	2000
$T_{hmtp}$	4.398
$T_{epm}$	$4.46\times 10^{-6}$
$T_{epa}$	6.552
$T_{h}$	2.294
$T_{RSSI}$	11.072 ^a^
$T_{ecc-sign}$	3460
$T_{ecc-verify}$	7634

^a^ $T_{RSSI}=$ 2.649 × 4 + 0.1584 × 2 + 0.0272 × 4 + 0.0486 = 11.072 μs.

**Table 4:** Comparisons of the execution time of five schemes.

Method	Signing a Single Message (μs)	Verify a Single Message (μs)	Verify *n* Messages (μs)
Giorgio’s scheme	$T=T_{ecc-sign}=3460$	$T=T_{ecc-verify}=7634$	$T={nT}_{ecc-verify}=7634n$ ^a^
Shim’s scheme	$T=2T_{epm}+T_{epa}$ $+T_{h}=8.6$	$T{=3T}_{bp}+T_{epa}+2T_{epm}+{2T}_{h}=6011$	$T={3T}_{bp}+\left(3n-2\right)T_{epa}+\left(n+1\right)T_{epm}+{2nT}_{h}=24.2n+5986.6$
Lo’s scheme	$T=T_{h}+T_{epm}=2.3$	$T{=2T}_{h}{+3T}_{epm}+2T_{epa}=17.7$	$T=2{nT}_{h}+2nT_{epa}+\left(n+2\right)T_{epm}=17.7n$
Horng’s scheme	$T{=T}_{h}+4T_{epm}{+T}_{epa}+2T_{hmtp}=17.64$	$T=2T_{bp}+T_{h}+T_{epa}+2T_{epm}+T_{hmtp}=4013.2$	$T={2T}_{bp}+{nT}_{h}$ $+\left(3n-1\right)T_{epa}+{3nT}_{epm}$ $+{nT}_{hmtp}=26.3n+3993.5$
Our scheme	Vehicle: $T=T_{h}+T_{epm}=2.3$	$T=2T_{h}+3T_{epa}+3T_{epm}=24.2$	$T{=2nT}_{h}+3nT_{epa}$ $+\left(n+2\right)T_{epm}=24.2n$
	RSU: $T{=T}_{RSSI}+T_{h}+T_{epm}=13.4$		

^a^ n is the number of messages.

**Table 5:** Communication costs of the proposed scheme.

**Communication Costs for a Vehicle (bit)**	PID	$P_{1}$	$P_{2}$	M	L	v	Timestamp	T	-
	256	512	512	256	512	256	32	32	-
**Communication Costs for a RSU (bit)**	$\left(x_{R}^{\prime },y_{R}^{\prime }\right)$	${ID}_{R2}^{\prime }$	$P_{R1}$	$P_{R2}$	M	$L_{R}$	Timestamp	t	$v_{R}$
	32	256	512	512	256	512	32	32	256

**Table 6:** Comparison of communication costs.

Method	Communication Overhead	After Reduction (byte)
**Shim’s Scheme**	512 + 512 + 32 + 256 + 32 + 512 + 512 + 512 = 2880 bits = 360 bytes	232
**Lo’s Scheme**	512 + 512 + 32 + 256 + 32 + 512 + 512 + 256 = 2624 bits = 328 bytes	232
**Horng’s Scheme**	512 + 512 + 512 + 256 + 512 = 2304 bits = 288 bytes	224
**Our Scheme**	For a vehicle: 296 bytes	200
	For a RSU: 300 bytes	204
